# 557 Current Assessment and Evaluation of Burn-Induced Chronic Pain

**DOI:** 10.1093/jbcr/irae036.191

**Published:** 2024-04-17

**Authors:** Chloe E Chapin-Grabowski, Victoria Marshall

**Affiliations:** Tampa General Hospital, Tampa, FL; University of South Florida, Tampa, FL; Tampa General Hospital, Tampa, FL; University of South Florida, Tampa, FL

## Abstract

**Introduction:**

Examine the current state of the literature regarding how chronic burn-induced pain is assessed and evaluated.

**Methods:**

Whittemore and Knafl’s integrative methodology was utilized to guide this literature review. A search of Pubmed, CINAHL, and PsycInfo resulted in nine articles included in the final synthesis. Inclusion criteria consisted of peer-reviewed studies in the English language published within the last five years. No restriction was placed on the type of burn or degree of injury. Reviewing how chronic burn-related pain is conceptualized precludes treatment modalities therefore, intervention-based studies were excluded. In addition, studies that did not evaluate long-term burn pain as a primary outcome measure were also excluded.

**Results:**

Chronic burn pain is most often assessed using numerical/visual analog scales and Likert scales. Occasionally, patients are asked to self-report characteristics they identified as chronic burn pain. The instruments used to evaluate pain are administered by various methods including pen-and-paper assessments, interviews, mail-in surveys and retroactive chart reviews. Studies assessed chronic burn pain at both a one-time basis and at intervals commencing as early as three months and extending as long as eight years post injury. Additionally, comorbid, psychological, and physical factors were found to be associated with the development of chronic burn pain.

**Conclusions:**

Chronic pain-related complaints remain prevalent amongst the burn population. However, further studies are warranted to assess the reliability of measuring pain via numerical/ visual analog scales and Likert scales in the burn population. Chronic burn pain is recognized as a major influence on health-related quality of life outcomes with current research prioritizing the identification of predictive risk factors.

**Applicability of Research to Practice:**

Early interventions and the implementation of preventative measures upon the recognition of risk factors associated with the development of chronic burn pain is critical. Increased use of multi-dimensional instruments can provide a comprehensive understanding of the various dimensions chronic burn pain. Finally, in depth assessments and evaluations of chronic burn pain can contribute to the development of integrative care and lead to improved long-term outcomes for the burn population. Improved patient outcomes include prioritizing burn survivors’ psychological well-being, providing individualized care for various classifications of burn injuries and differentiating neurogenic and neuropathic pain experiences.

